# Transmembrane signaling on a protocell: Creation of receptor-enzyme chimeras for immunodetection of specific antibodies and antigens

**DOI:** 10.1038/s41598-019-54539-7

**Published:** 2019-12-03

**Authors:** Jiulong Su, Tetsuya Kitaguchi, Yuki Ohmuro-Matsuyama, Theresa Seah, Farid J. Ghadessy, Shawn Hoon, Hiroshi Ueda

**Affiliations:** 1 Graduate School of Life Science and Technology, Tokyo Institute of Technology, 4259-R1-18 Nagatsuta-cho, Midori-ku, Yokohama, Kanagawa 226-8503 Japan; 2 Laboratory for Chemistry and Life Science, Institute of Innovative Research, Tokyo Institute of Technology, 4259-R1-18 Nagatsuta-cho, Midori-ku, Yokohama, Kanagawa 226-8503 Japan; 3 Molecular Engineering Laboratory, Biomedical Sciences Institutes, Agency for Science Technology and Research (A*STAR), 61 Biopolis Drive, Singapore, 138673 Singapore; 4 p53 Laboratory, Agency for Science Technology and Research (A*STAR), 8A Biomedical Grove, Singapore, 138673 Singapore

**Keywords:** Synthetic biology, Immunological techniques, Biosensors

## Abstract

It is known that digital counting of fluorescent signals generated in many small compartments can significantly improve the detection sensitivity of the enzyme-linked immunosorbent assay (ELISA). However, the reported digital ELISA systems need extensive washing steps to remove background signal, which hampers their performance. To tackle this problem, we developed a vesicle (Protocell) array wherein binding of an external protein analyte is coupled to signal amplification and intra-vesicular fluorescence readout. We chose β-glucuronidase (GUS) as a reporter enzyme as its function requires assembly of four subunits through dimerization of a pair of dimers that can be inhibited by a set of interface mutations. Using a thermostabilized GUS mutant IV-5, we screened out an interface mutant (M516K, F517W) to create IV5_m_ - a mutant with high thermostability and activity conditional on induced dimerization. After tethering a short N-terminal tag and transmembrane (TM) sequences, the fusion protein was expressed by cell-free protein synthesis inside protocells. When a corresponding tag-specific antibody was applied outside of the protocells, a clear increase in GUS activity was observed inside vesicles by adding fluorescent substrate, probably due to spontaneous integration of the tagged TM protein into the vesicles and dimerization by the antibody bound to the displayed tag. Furthermore, using flow cytometry, quantitative digital read out was obtained by counting fluorescent protocells exposed to varying concentrations of external antibodies that included Trastuzumab. Additionally, through use of an anti-caffeine V_HH_-SpyCatcher fusion protein, caffeine could be detected using SpyTag-fused TM-IV5_m_ protein expressed in protocells, suggesting utility of this platform for detection of diverse antigen types.

## Introduction

The immunoassay can detect various targets by antigen-antibody interaction with high sensitivity and specificity. Compared with traditional analytical methods such as spectroscopy and chromatography, the immunoassay is faster and more convenient^1^. Many types of immunoassays such as sandwich enzyme-linked immunosorbent assay (ELISA) have been widely used^2^. Recently, ELISA systems performed on microchambers to give digital counting of binding signals were reported to significantly increase the detection sensitivity^3,4^. However, like the traditional ELISA method, extensive washing steps are also needed for this digital ELISA system to remove background signal, which hampers its performance. Meanwhile, protocell arrays have been deployed for sensitive DNA analysis that provides a digitized signal^5,6^. Creation of a protocell array that amplifies the binding signal of external protein as a fluorescent signal of individual protocell will be a powerful clue to tackle this drawback of digital ELISA.

A variety of model membranes have been developed to construct bilayer vesicles in different sizes. Among them giant unilamellar vesicles (GUVs), referred to here as protocells, have a diameter larger than 1 μm and are ideal vesicles for an array system due to their similar size to cells and absence of organelles^7^. Protocells formed by inverted emulsion system and incorporated with the *in vitro* protein translation system have proved to be efficient in synthesized functional proteins such as green fluorescence protein (GFP)^8^ and transmembrane proteins^9,10^.

**Figure 1:**
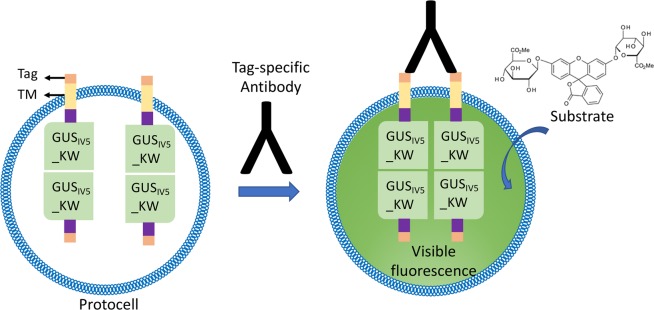
Scheme depicting detection of tag-specific antibodies using engineered protocells. External binding of a bivalent target such as antibody results in intra-vesicular enzyme dimerization and signal generation. To facilitate display of TM-fused subunit, non TM-fused subunit was co-expressed.

In natural cells, extracellular ligand binding signal is usually transduced by transmembrane receptors, and in many cases, dimerization of the receptor intracellular domain triggers activation of enzymes including kinases and subsequent signaling cascade. However, reconstruction of natural signaling cascades to get reliable signal in individual protocells is considered difficult. To mimic such natural signaling, here we used mutant beta-glucuronidase (GUS) as an alternative signal generator. GUS is a self-assembling tetrameric enzyme that catalyzes breakdown of complex carbohydrates. The tetramer state is necessary for the activity of GUS^11^ and can be prevented by a set of interface mutations^12^. Previously, a thermostabilized mutant of GUS (GUS_IV5_)^13^ was used to screen out a set of interface mutations (M516K, F517W) to give GUS_IV5__KW which shows high activity when tetramerized and low background at the inactive dimer state^14^. In order to transduce an external ligand binding event to generate intra-protocellullar signal, the transmembrane (TM) sequence from human epidermal growth factor receptor (EGFR)^15^ with epitope tags on its N-terminal was tethered to GUS_IV5__KW to make fusion proteins with membrane spanning capability capable of generating a ligand-dependent fluorescence signal (Fig. 1).

As the external targets, we first chose several commonly used anti-tag antibodies. The bivalent nature of these IgG antibodies is expected to dimerize the two membrane-exposed tag sequences, which will drive the association of tethered GUS_IV5_-KW domains inside protocells. Secondly, in view of practical application in therapeutic drug monitoring (TDM), we tried to detect Trastuzumab, a human anti-Her2 antibody using a mimotope sequence^16^ instead of epitope. Finally, to expand the scope of this protocell system, we employed SpyCatcher-SpyTag technology^17^ to prepare a nanobody (V_HH_)-fused SpyCatcher protein, and applied it to SpyTag-displaying protocells for detection of the membrane impermeable small antigen caffeine.

## Results

### Display of His-tag on the surface of protocell membrane

We first chose His-tag (HHHHHH) as a model epitope because of its short length and moderate hydrophobicity. To make protocells that display His-tag on their surface and transmit antibody-mediated dimerization signal into their interior, His_6_-TM-GUS_IV5__KW protein was synthesized by *in vitro* transcription/translation using a cell-free translation system with pure components (PUREfrex® 1.0) in protocells prepared by inverted emulsion method as described in the experimental section. We expected that the short tag sequence immediately after the N-terminal methionine of synthesized fusion protein can spontaneously traverse the lipid bilayer, and be displayed on the outer membrane surface with the aid of EGFR TM. To confirm the display of His_6_ tag, we incubated the recovered protocells serially with biotin-conjugated anti-His_6_ antibody and streptavidin-phycoerythrin (PE) (Supplementary Fig. S1a). After washing the excess dye, protocells labeled with PE were clearly observed under the fluorescence microscope. On the contrary, no fluorescence was observed when no antibody was used (Supplementary Fig. S1b). Hence, the N-terminal His_6_-tag was confirmed to be displayed on the surface of protocell, revealing the spontaneous integration of TM domain of the fusion protein into protocell membrane.

### Qualitative detection of tag-specific antibodies using protocells

**Figure 2:**
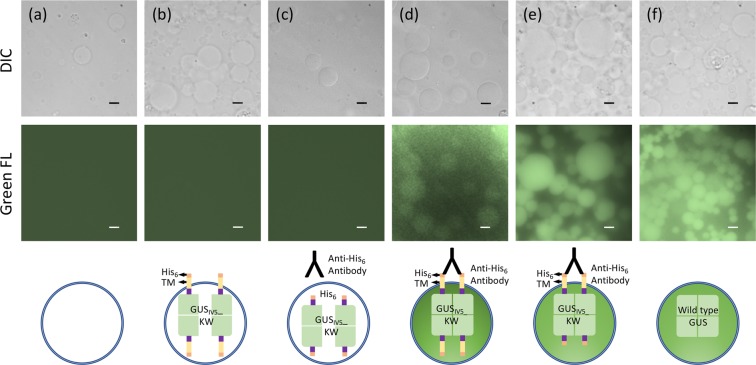
Differential interference contrast (DIC) (upper) and green fluorescence (lower) images of the protocells displaying His_6_-tag after substrate treatment. (**a**) Protocells without protein synthesized, (**b**) Protocells His_6_-TM-GUS_IV5__KW protein synthesized, (**c**) Protocells with His_6_-GUS_IV5__KW protein synthesized and treated with 0.1 μM anti-His_6_ antibody, (**d**) Protocells with His_6_-TM-GUS_IV5__KW protein synthesized and treated with 0.1 μM anti-His_6_ antibody, (**e**) Protocells with His_6_-TM-GUS_IV5__KW and His_6_-GUS_IV5__KW proteins synthesized together and treated with 0.1 μM anti-His_6_ antibody, (**f**) Protocells with wild type GUS synthesized. Scale Bars: 10 µm.

To attain transmembrane signaling by the dimerization of extracellular tag sequences, His_6_-TM-GUS_IV5__KW protein was synthesized alone or co-synthesized with His_6_-GUS_IV5__KW protein without TM in an equimolar amount by controlling the template concentration used for PUREfrex reaction to form GUS_IV5__KW dimers inside protocells (Fig. 1). After isolation of protocells with expressed fusion proteins and adding membrane-permeable fluorogenic substrate fluorescein di-β-d-glucuronide (FDGlcU) dimethyl ester, fluorescein generated by the enzyme reaction gave the protocell visible green fluorescence under microscopy (Fig. 2). Since the cleavage of substrate by GUS liberates more hydrophilic carboxyfluorescein, it was considered to remain within the protocells. As negative and positive controls, protocells with no synthesized protein (Fig. 2a) and with the wild-type GUS synthesized (Fig. 2f) were taken, respectively. Remarkably, after adding anti-His_6_ antibody to the protocells expressing His_6_-TM-GUS_IV5__KW protein and incubation for 30 min, a clear antibody-dependent fluorescence of the protocells was observed, probably reflecting the activation of GUS activity due to the dimerization of dimeric fusion proteins induced by antibody binding (Fig. 2b,d). Interestingly, the protocells co-expressing His_6_-TM-GUS_IV5__KW and His_6_-GUS_IV5__KW (Fig. 2e) showed even stronger fluorescence than the protocells expressing His_6_-TM-GUS_IV5__KW alone, probably due to membrane-free His_6_-GUS_IV5__KW protein in the protocell that could preferentially form a dimer with His_6_-TM-GUS_IV5__KW bound on the membrane without unfavorable entropic loss, and could give more stable active tetramers after antibody binding. As another negative control, the protocells expressing TM-free His_6_-GUS_IV5__KW protein alone did not show detectable fluorescence even in the presence of anti-His_6_ antibody (Fig. 2b). This clearly shows the necessity of a TM sequence for the display of His tag on the protocell. Considering the higher display efficiency of TM-encoding fusion proteins, in the following experiments, His_6_-GUS_IV5__KW without TM sequence was always co-synthesized with transmembrane fusion proteins to obtain better fluorescence signal.

**Figure 3:**
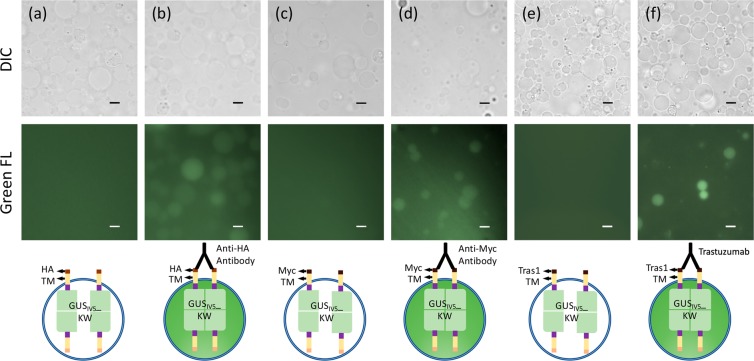
DIC (upper) and green fluorescence (lower) images of the protocells displaying HA-tag, myc-tag, and anti-Her2 mimotope. (**a,b**) Protocells with HA-TM-GUS_IV5__KW and His_6_-GUS_IV5__KW proteins synthesized, and treated with 0.1 μM anti-HA antibody in (**b**), (**c,d**) Protocells with Myc-TM-GUS_IV5__KW and His_6_-GUS_IV5__KW proteins synthesized, and treated with 0.1 μM anti-myc antibody in (**d**). (**e,f**) Protocells with Tras1 mimotope-TM-GUS_V5__KW and His_6_-GUS_IV5__KW proteins synthesized, and treated with 1 μM Trastuzumab in (**f**). Scale Bars: 10 µm

To show the generality of anti-tag antibody detection by the tag-displaying protocells, HA (YPYDVPDYA) and Myc (EQKLISEEDL) tags were displayed similarly on the protocell surface by constructing and synthesizing HA-TM-GUS_IV5__KW and Myc-TM-GUS_IV5__KW proteins together with His_6_-GUS_IV5__KW protein using the same method. After applying anti-HA antibody and anti-Myc antibody in the extravesicular solution, respectively, fluorescence caused by the antibody-induced enzyme activity was clearly observed under fluorescence microscopy (Fig. 3a–d). The result revealed the successful display of these short tags and the conversion of extravesicular antibody binding signal to intra-protocellular fluorescence signal.

### Quantitative detection of anti-His_6_ antibody using protocells

**Figure 4:**
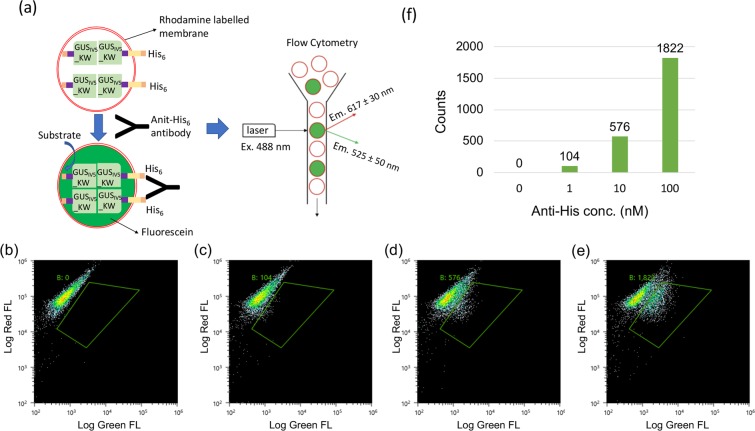
Digital detection of anti-His antibody. (**a**) Scheme of flow cytometric analysis for protocells. (**b–e**) Flow cytometric analysis for protocells displaying His_6_-tag responding to anti-His_6_ antibody in gradient concentrations of 0 nM (**b**), 1 nM (c), 10 nM (**d**) and 100 nM (**e**). (**f**) Event counts for the GreenFL-positive protocells in the selected region at the respective anti-His_6_ antibody concentration.

To quantify the fluorescence signal and measure the extravesicular antibody dose-response, protocells labeled with rhodamine-DHPE which expressed His_6_-TM-GUS_IV5__KW and His_6_-GUS_IV5__KW proteins were incubated with anti-His_6_ antibody in gradient concentrations for 30 min at room temperature, and analyzed by flow cytometry after incubation with the substrate for 30 min (Fig. 4a). The green fluorescence showing the GUS activity and the red fluorescence showing the size of individual protocells were then plotted to discriminate GUS-positive protocells (Fig. 4b–e). A region was set so that when no antibody was added, the population of protocells showing higher enzyme activity than background was zero, and the same region was used for counting fluorescein-positive cells, throughout a series of measurements. When the antibody concentration was increased, the counts of protocells showing higher enzyme activity increased correspondingly. By obtaining the event counts of the fraction with antibody induced enzyme activity, a digital signal corresponding to antibody concentration was obtained (Fig. 4f).

### Quantitative detection of trastuzumab using protocells

To investigate the possible use of this system in TDM, a Trastuzumab mimotope sequence (TRAS-1: QLGPYELWELSH)^16^ was cloned into the N-terminal of TM-GUS_IV5__KW sequence, and quantitative detection of Trastuzumab was tested similarly to anti-His tag antibody. To increase the fluorescent signal, we tried to increase protocell membrane stability by altering membrane composition through addition of PEG2000PE and more cholesterol^18^, and extended the incubation time to 3 h. After applying Trastuzumab in the extravesicular solution, fluorescence caused by the antibody-induced enzyme activity was clearly observed under fluorescence microscopy (Fig. 3e–f). Also, when the protocells incubated with varying concentrations of Trastuzumab were analyzed by FCM, similar results to anti-His antibody detection were obtained (Supplementary Fig. S2). It is worth noting that compared with the original bioluminescence resonance energy transfer-based sensor (TRAS-LUMABS-1)^16^, a lower concentration of 10 nM Trastuzumab could be detected.

### Detection of dimerized SpyCatcher protein by protocells displaying SpyTag

**Figure 5:**
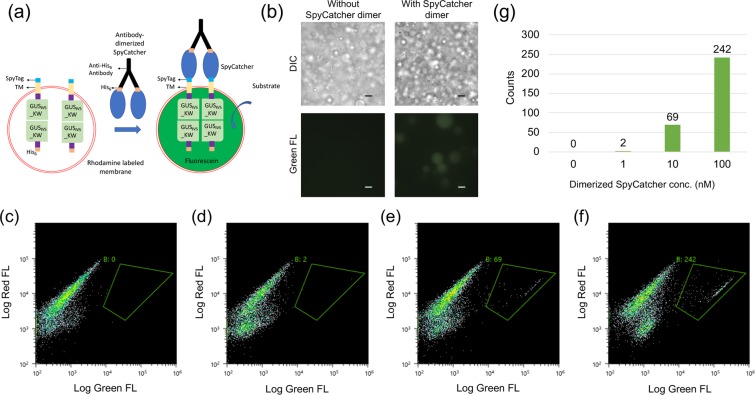
Detection of SpyCatcher dimers. (**a**) Scheme for protocells displaying SpyTag activated by antibody-dimerized SpyCatcher binding. (**b**) DIC (upper) and fluorescence (lower) microcopy of stabilized protocells without rhodamine-DHPC displaying SpyTag, with and without addition of 0.1 μM SpyCatcher dimer after 3 h incubation with substrate. Scale Bars: 10 µm. (**c–f**) Flow cytometric analysis for protocells displaying SpyTag responding to SpyCatcher dimer at 0 nM (**c**), 1 nM (**d**), 10 nM (e), and 100 nM (**f**). (**g**) Event counts for the positive protocells at the respective SpyCatcher dimer concentration.

SpyTag/SpyCatcher is a powerful covalent isopeptide conjugation system for bioconjugation and engineering protein architectures^17,19^. To test the possibility of applying SpyTag/SpyCatcher system as a connector between the protocell biosensor and an extracellular binding domain such as an antibody fragment, an expression vector for SpyTag-TM-GUS_IV5__KW was constructed. This protein and His_6_-GUS_IV5__KW without TM were next synthesized inside protocells with improved membrane composition as in the Trastuzumab detection. To investigate the functional ligation of SpyTag/SpyCatcher, His_6_-SpyCatcher protein was expressed in *E. coli* (Supplementary Fig. S3) and dimerized by anti-His_6_ antibody (Fig. 5a**)**. After SpyCatcher dimers in different concentrations were incubated with protocell carrying SpyTag-TM-GUS_IV5__KW protein for 3 h at room temperature, microscopic observation and flow cytometric analysis were performed to measure the size and enzyme activity of protocells as previously described. As a result, SpyTag-displaying protocells incubated with SpyCatcher dimers were clearly observed under fluorescence microscopy (Fig. 5b**)** and the counts of protocells showing enzyme activity caused by GUS tetramerization increased correspondingly to the concentration of SpyCatcher dimers (Fig. 5c–f). From the protocell counts of the region with antibody induced enzyme activity, a quantified signal corresponding to antibody concentration can be obtained (Fig. 5g). The lower counts compared to His_6_-tag displaying protocells might indicate the lower display efficiency of SpyTag and/or lower efficiency of dimerization compared with direct His_6_-tag crosslinking, which might also result in difficulties observing fluorescent protocells using the original membrane composition by microscopy **(**Supplementary Fig. S4**)**. However, we could still perform FCM analysis of those original protocells with lower fluorescent intensities in 30 min **(**Supplementary Fig. S5**)**.

### Detection of caffeine using anti-caffeine V_HH_-SpyCatcher protein and protocells displaying SpyTag

Next, we employed a nanobody (V_HH_)-SpyCatcher fusion protein instead of His_6_-SpyCatcher protein to drive protocell signal transduction. Interestingly, an anti-caffeine V_HH_ was reported to show hapten-induced dimerization^14^. Based on this idea, an expression vector for adaptor protein consisting of anti-caffeine V_HH_ fused with SpyCatcher was made, and the protein expressed in *E. coli* SHuffle T7 express lysY and purified to homogeneity **(**Supplementary Fig. S6**)**.

**Figure 6:**
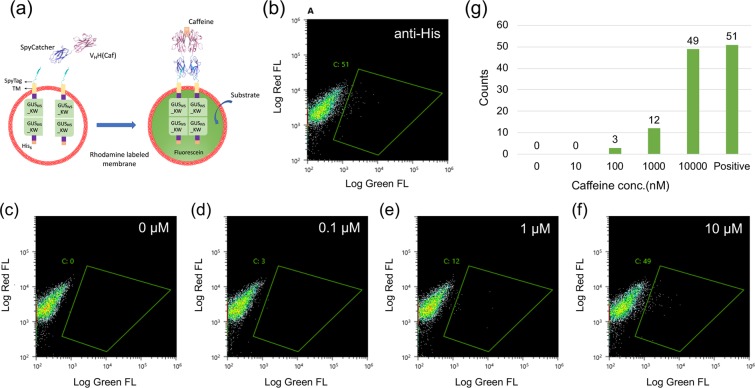
Detection of Caffeine. (**a**) Scheme for protocells displaying SpyTag activated by antigen-dimerized V_HH_-SpyCatcher. (**b**) Modified protocells displaying SpyTag with and without 0.1 μM SpyCatcher dimer after 3 h incubation with substrate. (**b–f**) Flow cytometric analysis for protocells displaying SpyTag responding to V_HH_-SpyCatcher dimer in the presence of 0.1 µM anti-His antibody (**b**) or 0 nM (**c**), 0.1 µM (**d**), 1 µM (**e**) and 10 µM (**f**) of caffeine. (**g**) Event counts for the positive protocells at the respective SpyCatcher dimer concentration.

V_HH_-SpyCatcher in the presence of different concentrations of caffeine or 0.1 µM anti-His antibody as a positive control was incubated with protocell carrying SpyTag-TM-GUS_IV5__KW protein for 3 h at room temperature. Flow cytometry was then used to measure the size and enzyme activity of protocells as before **(**Fig. 6**)**. The number of protocells showing enzyme activity caused by GUS tetramerization increased upon addition of anti-His antibody (Fig. 6b,c) and further corresponded to the caffeine concentration (Fig. 6c–f). From the counts of protocells in the region with increased Green FL, a quantified signal corresponding to caffeine concentration was obtained (Fig. 6g). Caffeine is present in many widely consumed beverages, and it can be toxic to adults at intake levels exceeding 500 mg per day^20^. Considering that the caffeine concentration of tea is approximately 140 μM and that in coffee is approximately 5 mM^21^, this system will be able detect caffeine in common foods and beverages.

## Discussion

In this study, a protocell-based biosensor system was constructed and successfully used for the detection of tag-specific antibodies and a small antigen that drives dimerization of its binding nanobody. Compared with other solution-based biosensor systems, the protocell increases sensitivity by decreasing the background signal and providing signals in an “on/off” manner. The simpler system than digital ELISA improved assay handlings; the protocell biosensor does not need any separation steps before measurement and permits assay in a one-step reaction. This gives the protocell system a great potential to serve as an efficient detection method. Furthermore, the successful detection of Trastuzumab indicates the system’s potential application in TDM. Compared with other enzyme-based biosensors, the proposed system has an additional merit that it is insensitive to possible intrinsic enzyme (GUS) activity in samples such as body fluid. Additionally, the positive responses to externally dimerized SpyCatcher by SpyTag displaying protocells will make it possible to detect various targets by fusing SpyCatcher with not only nanobodies but with many other binding domains, such as single chain antibody fragments.

To understand the detectability of a single activated enzyme tetramer in one protocell, which is considered necessary to obtain single molecule-derived digital signal^3^, the amount of final product of the enzyme reaction was calculated. Based on the turnover number *K*_cat_ of GUS_IV5__KW protein for the fluorescent substrate measured as 1.18 s^−1^ (Supplementary Fig. S7), it was calculated that in a protocell with 2 μm diameter containing one molecule of fully activated GUS_IV5__KW can give 45 nM fluorescein in this protocell after 1 h of reaction. If a more stable protocell is used to extend the reaction time to 3 h, 135 nM fluorescein will be obtained. When we prepared a set of protocells with varied concentrations of fluorescein inside and measured their signal by flow cytometry, the protocells with 100 nM fluorescein inside were easily detectable by this method (Supplementary Fig. S8). This means that the protocell biosensor system will be able to provide digital read outs by controlling the size and the amount of proteins synthesized.

By providing low background and a digitalized signal, sensitivity of this protocell biosensor system for use in immunodetection has been greatly increased. Considering the potential in detecting multiple targets and protein interactions, the developed protocell biosensor system will be very useful in immunological studies and medical diagnostic settings.

## Experimental

### Materials

1-Palmitoyl-2-oleoyl-sn-glycero-3-phosphocholine (POPC) was purchased from NOF corporation (Tokyo, Japan), cholesterol was from Nacalai Tesque (Kyoto, Japan), and 1,2-distearoyl-sn-glycero-3-phosphoethanolamine-N-[methoxy (polyethylene glycol)-2000] (PEG2000PE) was purchased from Avanti Polar Lipids (Alabaster, AL, USA). Biotin-conjugated anti-PentaHis antibody was purchased from Qiagen Japan (Tokyo, Japan). Streptavidin-PE was purchased from Miltenyi Biotec (Bergisch Gladbach, Germany). Rhodamine B 1, 2-dihexadecanoyl-sn-glycero-3- phosphoethanolamine, triethylammonium salt (rhodamine-DHPE) was purchased form Setareh Biotech (Eugene, OR, USA). PUREfrex 1.0 system was purchased from GeneFrontier (Chiba, Japan). The expression vector for His-Avi-tagged SpyCatcher002 (pDEST14-Avitag-SpyCatcher002) was obtained from Addgene (Watertown, MA, USA). The DNA encoding SpyTag002 was synthesized by Eurofin Genomics (Tokyo, Japan). *E. coli* SHuffle T7 Express lysY for protein expression and the restriction enzymes were obtained from NEB Japan (Tokyo, Japan). Monoclonal anti-His (28–75), anti-HA, and anti myc antibodies was from Fujifilm Wako. Other chemicals were purchased from Fujifilm Wako Chemicals (Tokyo, Japan).

The following oligonucleotides were synthesized by Eurofin Genomics, and used:

His_NdeI_Back: 5′-AAGGAGATATACATATGCACCATCATCATCATCAT-3′

His_HindIII _For: 5′-ACCGCCACCAAGCTTATGATGATGATGATGGTGCA-3′

TM_His _For: 5′-CATCCCAGTGGCGATATGATGATGATGATGGTGCA-3′

HA_NdeI_Back: 5′-AAGGAGATATACATATGTATCCGTATGATGTGCCG-3′

TM_HA _For: 5′-CATCCCAGTGGCGATCGCATAATCCGGCACATCA-3′

Myc_NdeI_Back: 5′-AAGGAGATATACATATGGAACAGAAACTGATTAGC-3′

TM_Myc_For: 5′-CATCCCAGTGGCGATCAGATCTTCTTCGCTAATCA-3′

TM_Tras1_For: 5′-CATCCCAGTGGCGATATGCGACAATTCCCACAATTCGTAAGG-3′

NdeTras1_Back: 5′-AAGGAGATATACATATGCAGTTAGGCCCTTACGAATTGTGGG-3′

Spy_NdeI_Back: 5′-AAGGAGATATACATATGGTGCCTACTATCGTGATG-3′

TM_Spy_For: 5′-CATCCCAGTGGCGATCTTGTAACGCTTGTAGGC-3′

TM_Back: 5′-ATCGCCACTGGGATGG-3′

TM_HindIII_For: 5′-CACCGCCACCAAGCTTCATGAAGAGGCCGATCCC-3′

VHH-SpyCatcher_Infus_Back: 5′-GGTAGCGCGGCCGCTATGGTAACCACCTTATC-3′

VHH-SpyCatcher_Infus_For: 5′-GTGGTGGTGCTCGAGTTAGCTACCACTGGATCC-3′

### Construction of plasmids

His_6_-tag, HA-tag, Myc-tag and SpyTag DNAs were amplified as primer dimers by using the primer pairs His_NdeI_Back/TM_His_For, HA_NdeI_Back/TM_HA_For, Myc_NdeI_Back/TM_Myc_For, NdeTras1_Back/TM_Tras1_For and Spy_NdeI_Back/TM_Spy_For using a thermal cycler. The sequence encoding the transmembrane domain (TM) of human EGFR was amplified by PCR using TM_Back and TM_HindIII_For primers and pCO12-EGFR (Riken Bioresource Center, Tsukuba, Japan) as a template. DNAs of tags were fused to TM DNA by overlap extension PCR to give His_6_-TM, HA-TM, Myc-TM, Tras1-TM and SpyTag-TM DNAs. In short, His_6_-TM, HA-TM, Myc-TM, Trans1-TM and Spy-TM sequences were inserted into GUS_IV5__KW vector linearized by *Nde*I and *Hin*dIII, using an In-Fusion™ HD cloning kit (Takara-Bio, Shiga, Japan) to construct His_6_-TM- GUS_IV5__KW, HA-TM- GUS_IV5__KW, Myc-TM- GUS_IV5__KW, Trans1- GUS_IV5__KW and SpyTag-TM- GUS_IV5__KW vectors. His_6_-tag DNA with *Nde*I and *Hin*dIII restriction enzyme sites was amplified by PCR using His_NdeI_Back and His_HindIII _For primers and inserted into linearized GUS_IV5__KW vector with same restriction enzyme sites to construct His_6_- GUS_IV5__KW vector. The expression vector for V_H_H(Caf)-SpyCatcher protein was constructed by replacing GUS_IV5__KW gene in pET32-V_H_H(Caf)- GUS_IV5__KW^14^ with SpyCatcher002 gene amplified from pDEST14-Avitag-SpyCatcher002 with primers VHH-SpyCatcher_Infus_Back and VHH-SpyCatcher_Infus_For.

### Preparation of protocells

The protocells were prepared based on the inverted emulsion methods described by K. Nishimura *et al*.^8^. POPC and cholesterol dissolved in chloroform (100 mg/ml) were mixed in a 9:1 weight ratio in 400 µl paraffin, and water in oil emulsion was made by vortex for 30 s with 20 µl PUREfrex 1.0 system reaction mixture including 10 ng/µl template DNA, 1 µl (40 unit) of RNase inhibitor (Nippon Gene, Toyama, Japan) and 330 mM sucrose. After layering the emulsion (400 µl) on 150 µl of outer buffer (100 mM HEPES, 280 mM potassium glutamate, 20 mM Mg(OAc)_2_, NTPs (3.75 mM ATP, 2.5 mM GTP, 1.35 mM CTP, 1.35 mM UTP), amino acids (0.3 mM Tyr, 0.3 mM Cys and 0.375 mM for other 18 amino acids), 15 mM creation phosphate, 330 mM glucose, 20 µg/ml RNase; pH = 7.6) in a microtube, protocells were formed by centrifuge at 18,000 × g for 30 min at 4 °C. Outer buffer containing protocells was collected by piercing the bottom of the tube using a syringe with 18 G × 1 1/2 inch needle.

### Detection of enzyme activity

The liposomal suspension was centrifuged at 18,000 × g for 30 min, the supernatant discarded, and 150 μl of the outer buffer was added to resuspend the liposomes. The liposome suspension was incubated for 2 h at 37 °C for protein synthesis. For flow cytometry measurement, 0.1% (w/w) rhodamine-DHPE was included in membrane components. For displaying Tras1 and SpyTag on the protocells, a membrane composition with POPC: cholesterol: PEG2000PE = 5.75: 4: 0.25 in molar ratio^18^ was also tested. To the liposome suspension, anti-tag antibody (0.1 μM) was added and incubated at room temperature for 30 min. Afterwards, 0.1 mg/ml fluorescein di-β-d-glucuronide (FDGlcU) dimethyl ester (Marker Gene Technologies, Eugene, OR, USA) was added as a substrate, and reacted for 30 min (or 3 h for modified protocells composed of POPC, cholesterol and PEG2000PE) at room temperature before microscopic and flow cytometric analyses.

### Microscopic observation

Microscopy was performed by an IX 71 inverted microscope (Olympus, Tokyo, Japan). Samples were observed under a 60x objective lens. Fluorescein was excited at 495–500 nm and emission at >505 nm was observed. Phycoerythrin-labeled protocells was excited at 545–580 nm and emission at >600 nm was observed.

### Flow cytometric analysis

Flow cytometric analysis was performed using an SH-800 cell sorter (Sony, Tokyo, Japan). Ten thousand events were measured in each flow cytometric analysis. Both rhodamine DHPE and fluorescein were excited at 488 nm. The emission of rhodamine was detected through a 617/30 nm bandpass filter as Red FL and emission of fluorescein was detected through a 525/50 nm bandpass filter as Green FL.

## Supplementary information

Supplementary Figures
