# Supplementary material for: Transmembrane signaling on a protocell: Creation of receptor-enzyme chimeras for immunodetection of specific antibodies and antigens
Source: Sci Rep. 2019 Dec 3;9:18189. doi: 10.1038/s41598-019-54539-7 (PMC6890649; doi:10.1038/s41598-019-54539-7)
Supplement: Supplementary file 1 — Supplementary Figures [file 41598_2019_54539_MOESM1_ESM.pdf]

Supporting information for

Transmembrane signaling on a protocell: Creation of receptor-enzyme chimeras for immunodetection of specific antibodies and antigens

Jiulong Su[1], Tetsuya Kitaguchi[2], Yuki Ohmuro-Matsuyama[2], Theresa Seah[3], Farid J. Ghadessy[4], Shawn Hoon[3] and Hiroshi Ueda[2*]

[1]*Graduate School of Life Science and Technology, Tokyo Institute of Technology, 4259-R1-18 Nagatsuta-cho, Midori-ku, Yokohama, Kanagawa 226-8503, Japan*
[2]*Laboratory for Chemistry and Life Science, Institute of Innovative Research, Tokyo Institute of Technology, 4259-R1-18 Nagatsuta-cho, Midori-ku, Yokohama, Kanagawa 226-8503, Japan*
[3]*Molecular Engineering Laboratory, Biomedical Sciences Institutes, Agency for Science Technology and Research (A*STAR), 61 Biopolis Drive, 138673, Singapore*
[4]*p53 Laboratory, Agency for Science Technology and Research (A*STAR), 8A Biomedical Grove, 138673, Singapore*

[*] Corresponding author: H.U. ueda@res.titech.ac.jp

Figure S1. Display of His_6-tag on protocell membrane S-2
Figure S2. Digital detection of Trastuzumab S-3
Figure S3. Expression and IMAC purification of His_6-tagged SpyCatcher002 S-4
Figure S4. SpyTag-displaying protocells with original and optimized compositions S-5
Figure S5. FCM analysis of SpyTag-displaying protocells with original lipid composition S-6
Figure S6. Expression and purification of V_HH(Caf)-SpyCatcher protein S-7
Figure S7. Calculation of specific activity S-8
Figure S8. FCM analysis of protocells incorporating fluorescein in gradient concentrations. S-9

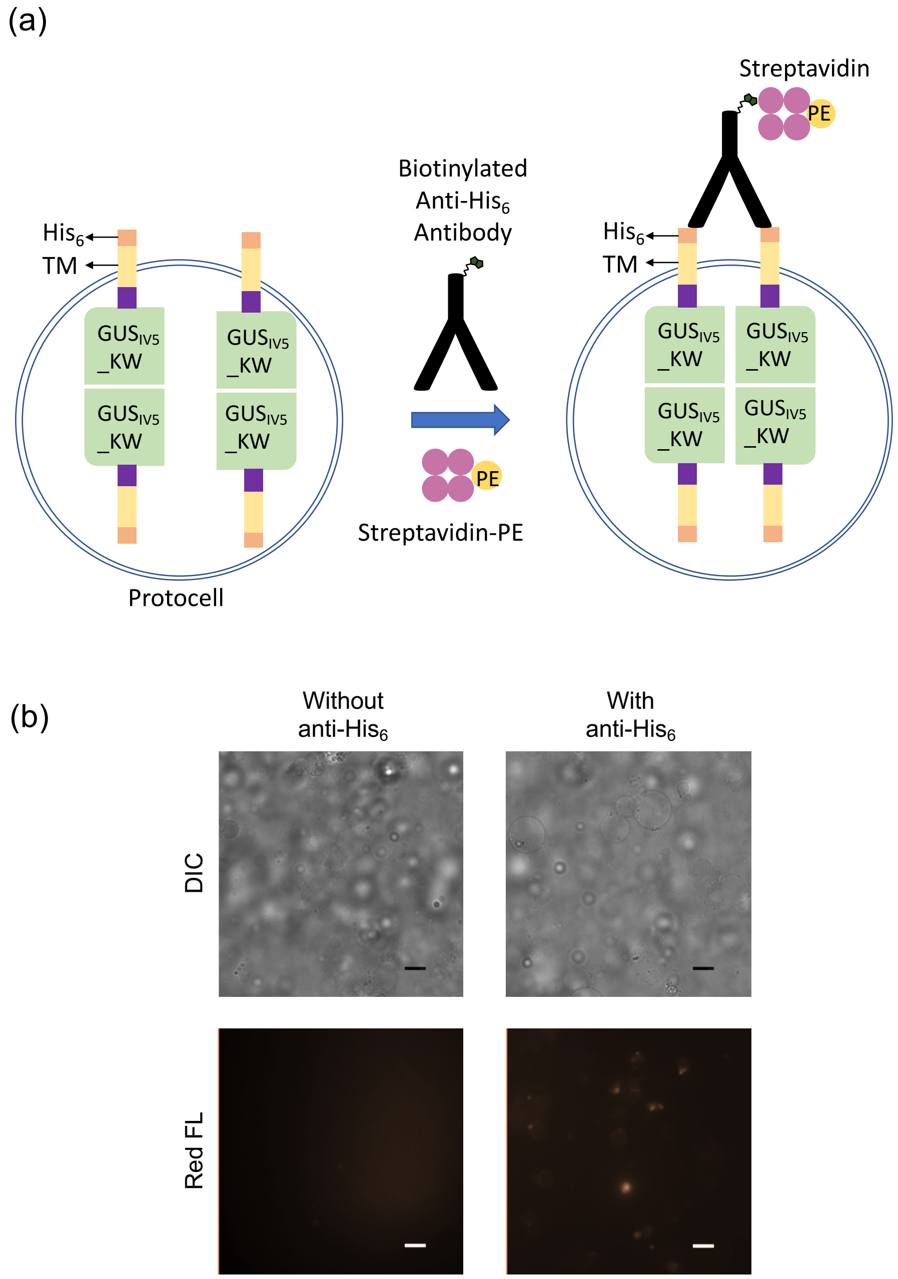

Figure S1. Display of His$_6$-tag on protocell membrane. (a) Scheme of His$_6$-tag display test. (b) Differential interference contrast (DIC) (upper) and red fluorescence (lower) images of the protocells incubated with (right) or without (left) treatment with biotinylated anti-His$_6$ antibody and Streptavidin-phycoerythrin (PE). Scale bar :10 μm.

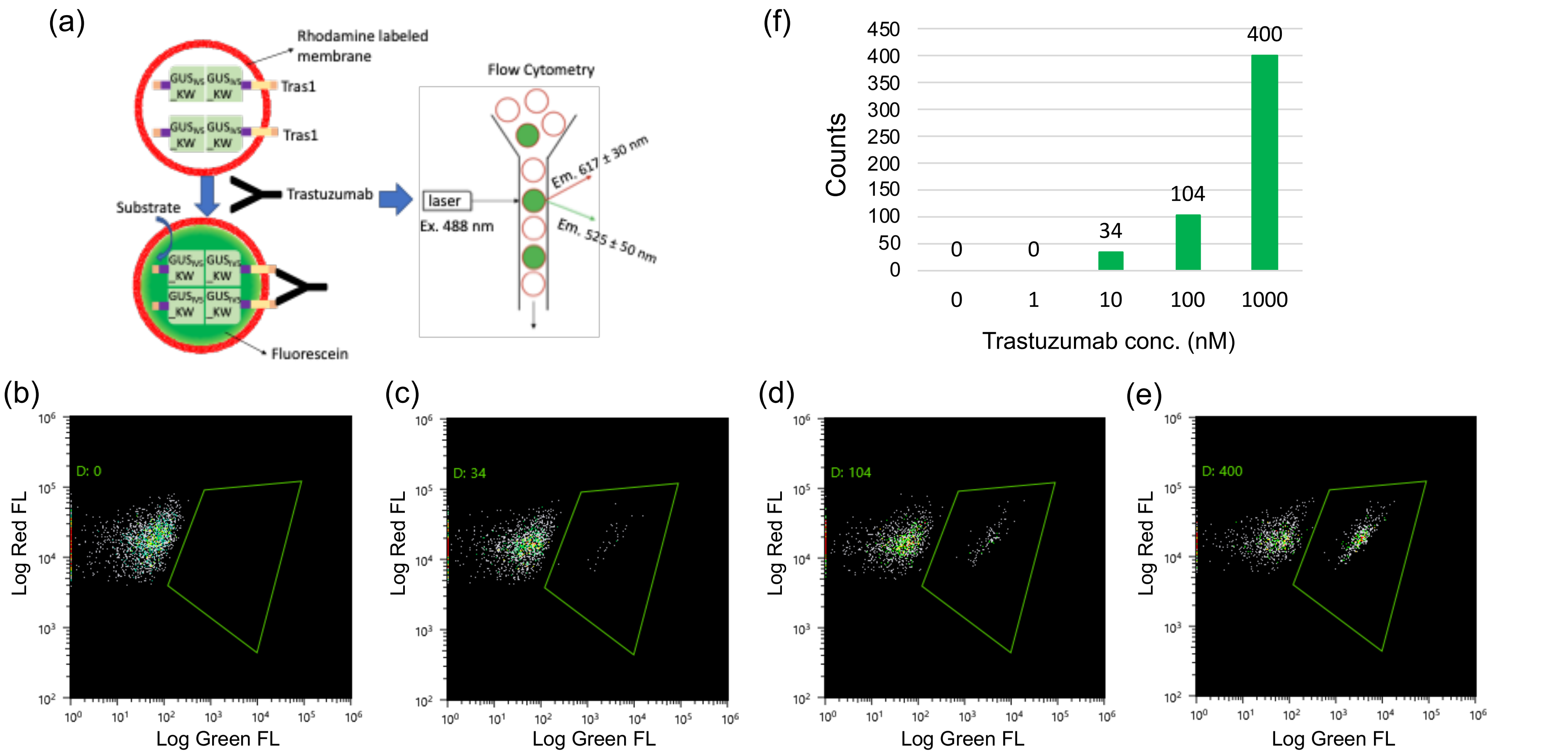

Figure S2. Digital detection of Trastuzumab. (a) Scheme of flow cytometric analysis for protocells. (b-e) Flow cytometric analysis for protocells displaying Tras1-tag responding to Trastuzumab in gradient concentrations of 0 nM (b), 10 nM (c), 100 nM (d) and 1 μM (e). (f) Event counts for Green-FL positive protocells at the respective Trastuzumab concentration.

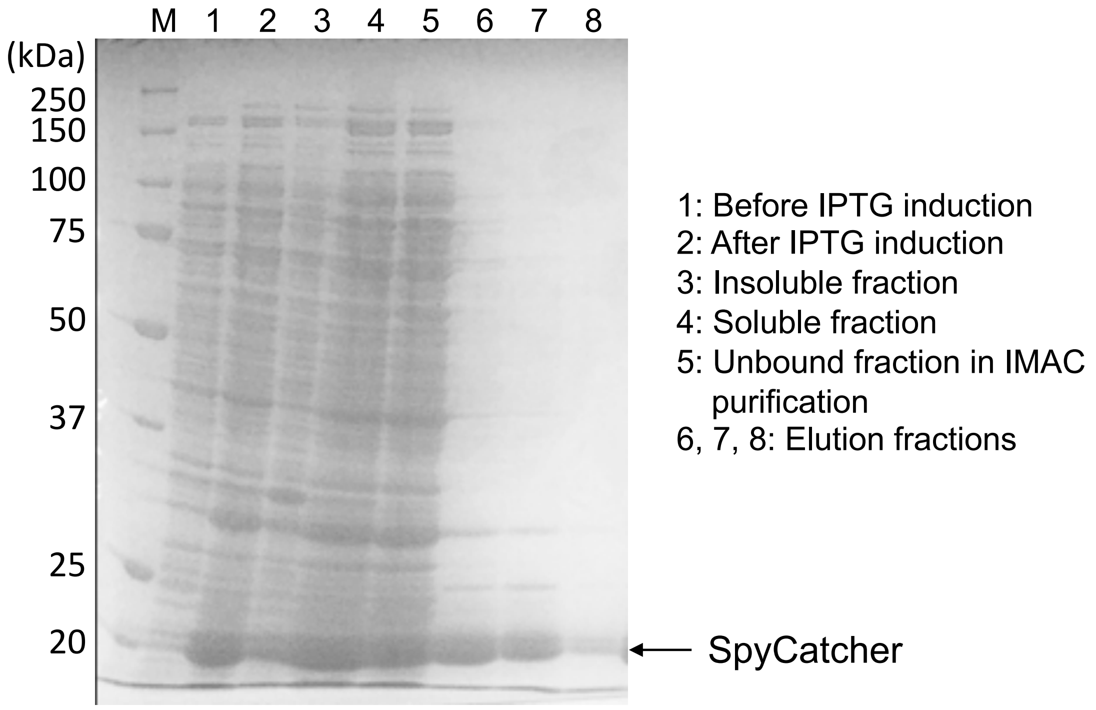

Figure S3. Expression and IMAC purification of His_6-tagged SpyCatcher002. The calculated molecular weight of the protein is 17.7 kDa. Some His-Avi-tagged SpyCatcher002 might be biotinylated at Avitag during expression in *E. coli* SHuffle T7 express lysY.

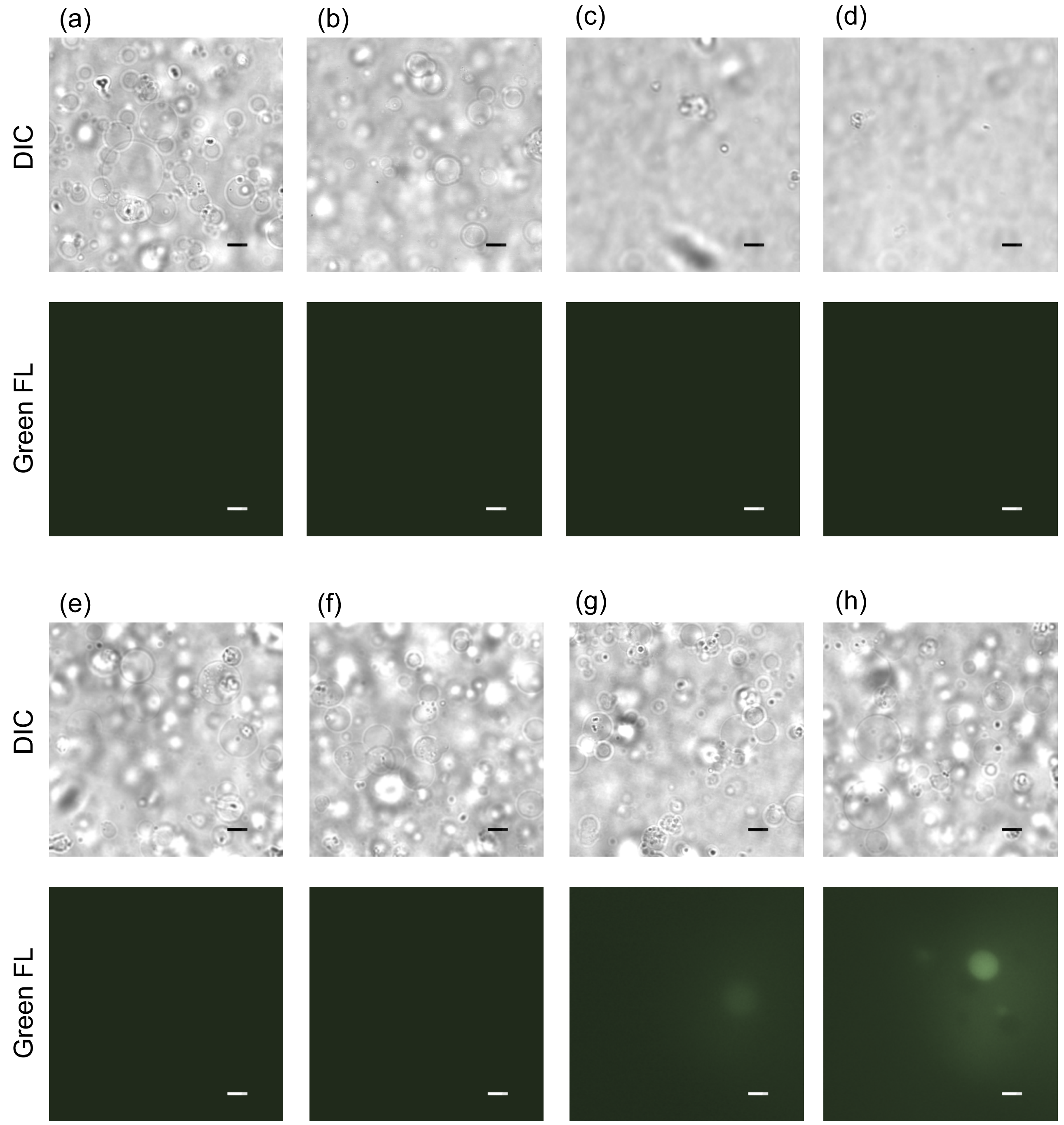

Figure S4. SpyTag-displaying protocells with original and optimized compositions. (a-d) Protocells of the original composition were incubated for 0 h (a), 1 h (b), 2 h (c) and 3 h (d) with the substrate. (e-h) Protocells of the optimized composition were incubated for 0 h (e), 1 h (f), 2 h (g) and 3 h (h) with the substrate (scale bar = 10 μm).

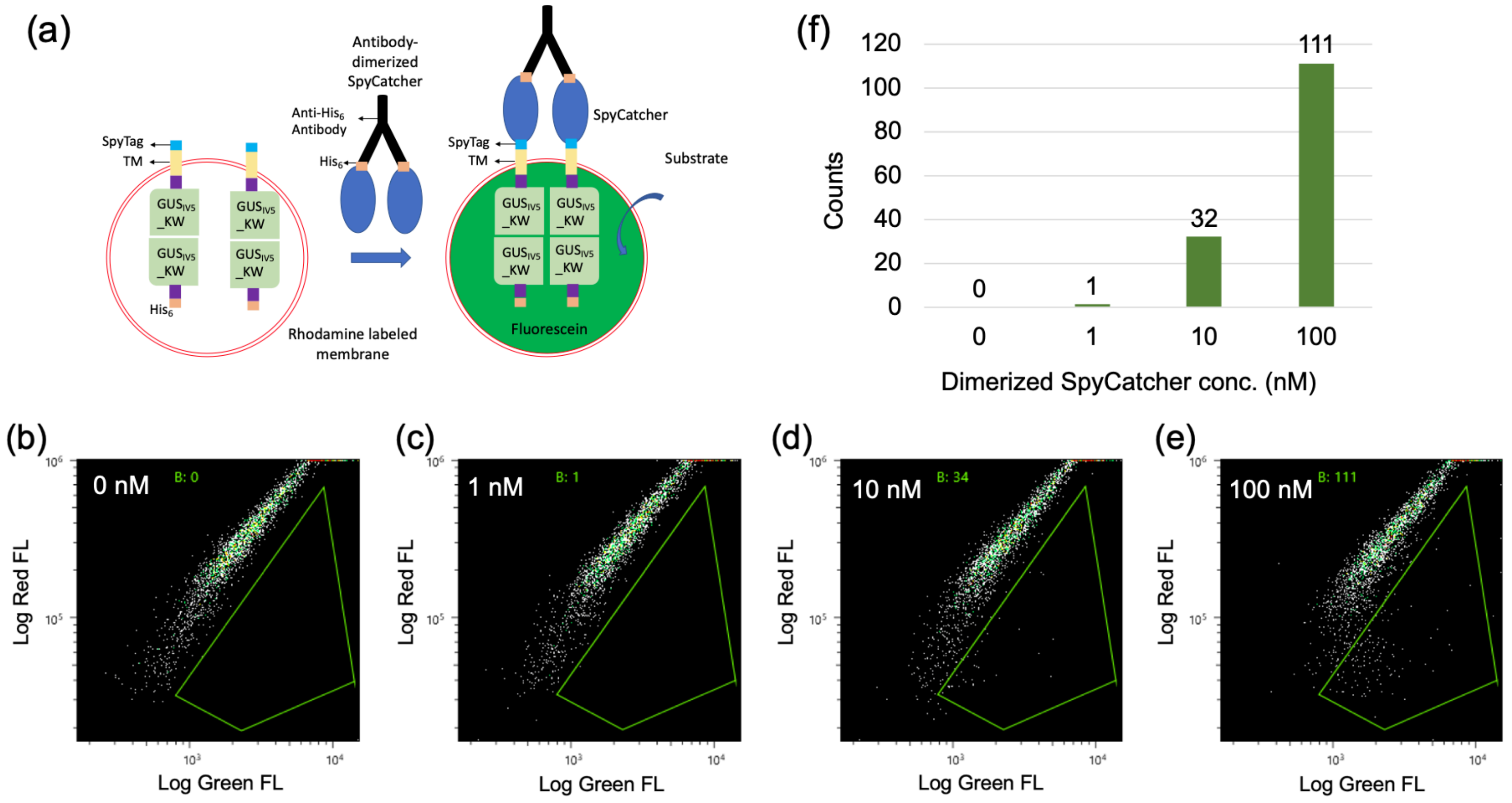

Figure S5. FCM analysis of SpyTag displaying protocells with original lipid composition. After 30 min incubation, responces to SpyCatcher dimers at gradient concentrations were analyzed.

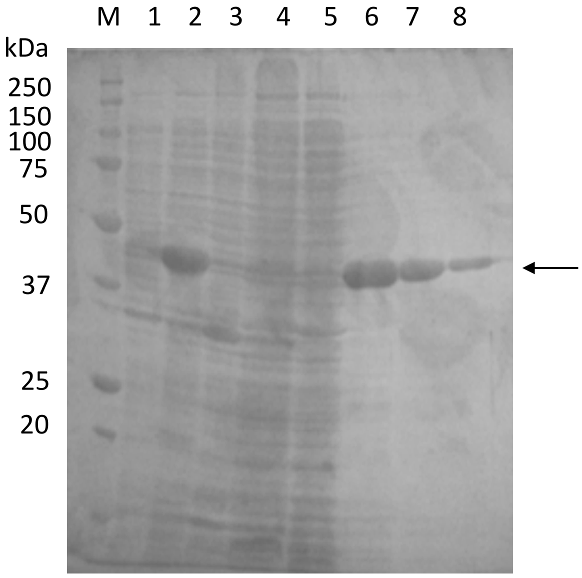

1: Before IPTG induction
2: After IPTG induction
3: Insoluble fraction
4: Soluble fraction
5: Unbound fraction in IMAC purification
6-8: Elution fractions of IMAC purification

Figure S6. Expression and purification of V_HH(Caf)-SpyCatcher protein (43 kDa).

(a)

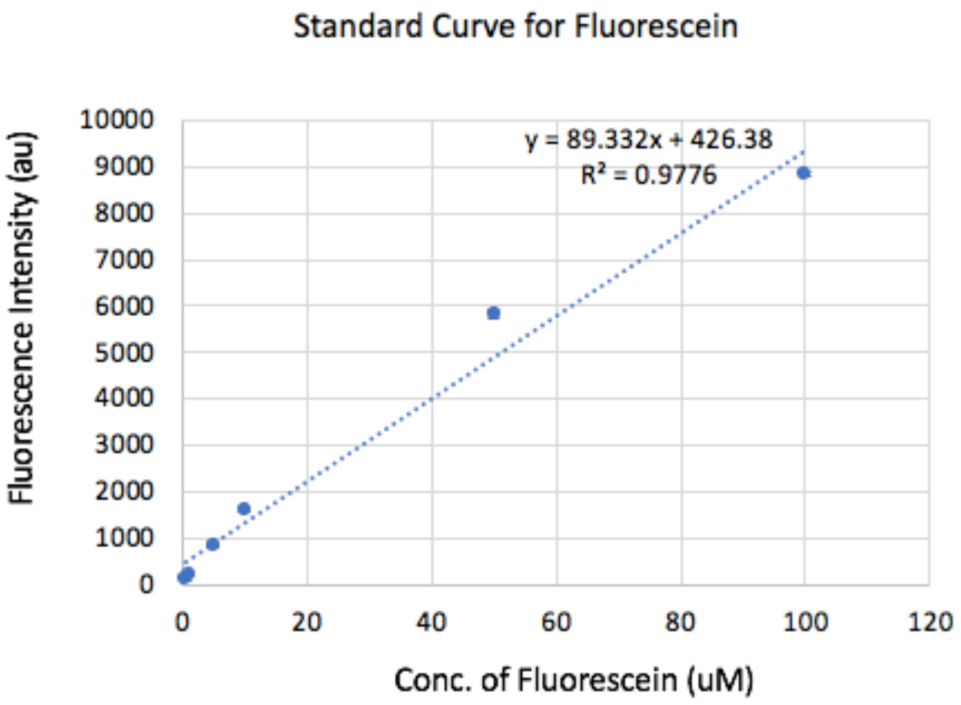

(b)

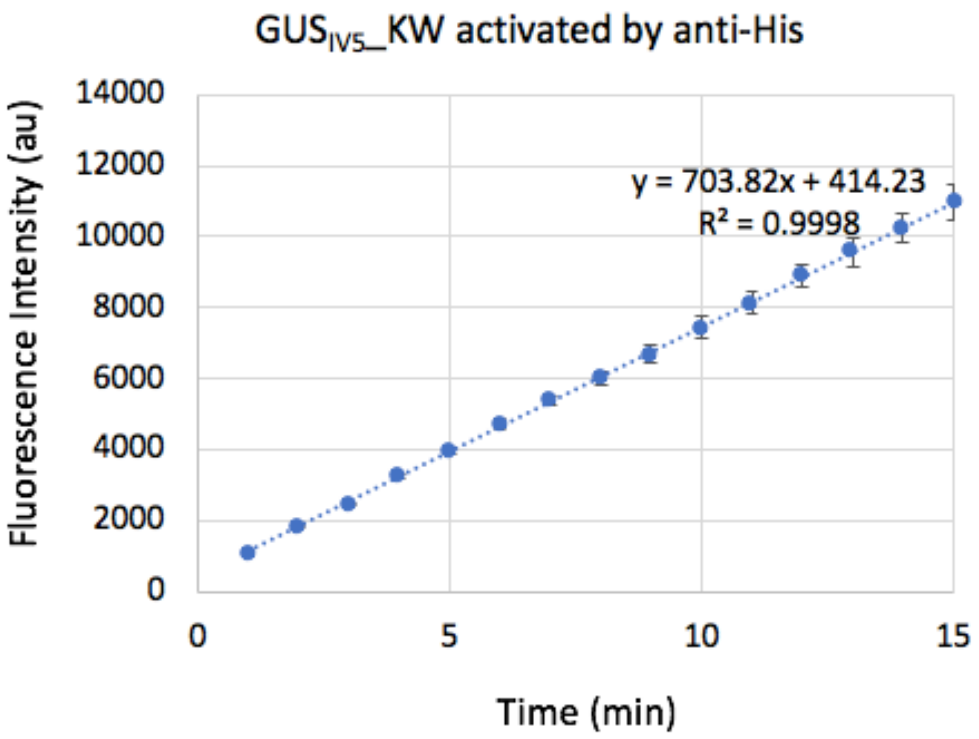

Figure S7. Calculation of specific activity. A standard curve of the final product fluorescein (a) and time course curve of GUS_{IV5}_KW tetramerized by anti-His antibody (b) were measured. Considering the protein concentration (0.1 μM), K_{cat} of GUS_{IV5}_KW was calculated as 1.18 s^{-1}

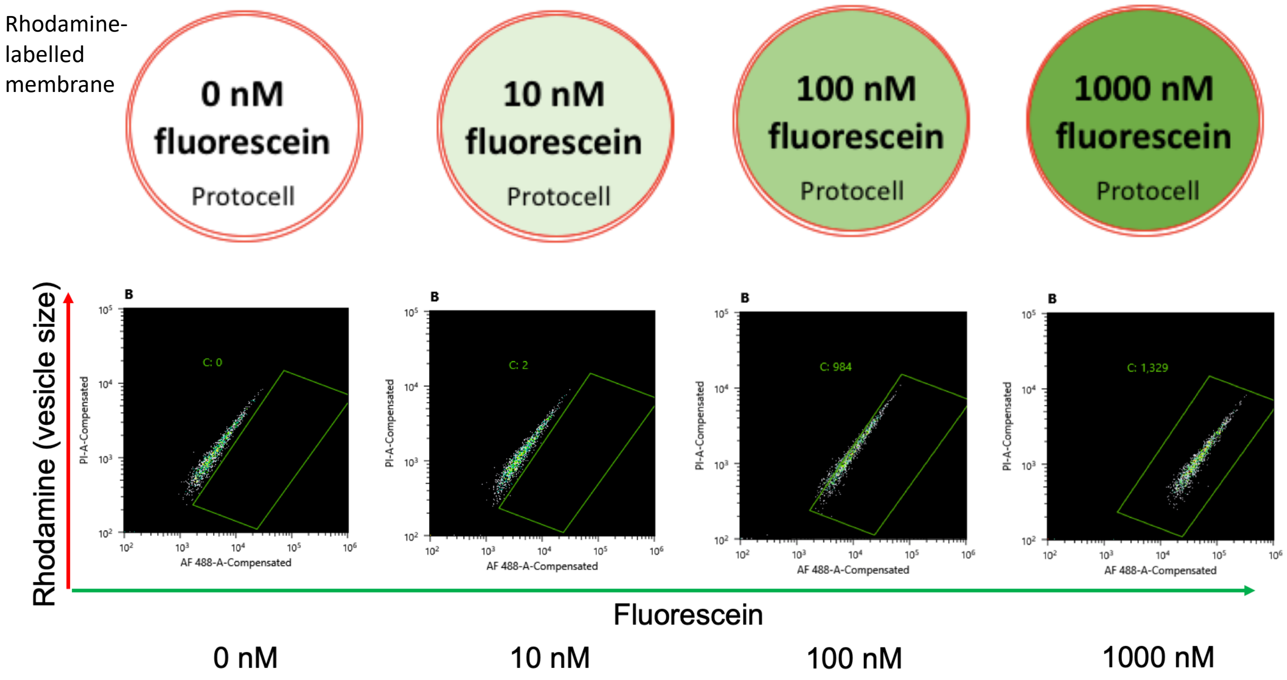

Figure S8. FCM analysis of protocells incorporating fluorescein in gradient concentrations.
